# Association of Histamine N-Methyltransferase Thr105Ile Polymorphism with Parkinson’s Disease and Schizophrenia in Han Chinese: A Case-Control Study

**DOI:** 10.1371/journal.pone.0119692

**Published:** 2015-03-13

**Authors:** Xinglong Yang, Chuanxin Liu, Jinxiang Zhang, Hongying Han, Xiuyan Wang, Zhoulin Liu, Yanming Xu

**Affiliations:** 1 Department of Neurology, West China Hospital, Sichuan University, 37 Guo Xue Xiang, Chengdu, Sichuan Province, 610041, PR China; 2 College of Basic and Forensic Medicine, Sichuan University, Chengdu, Sichuan Province, 610041, PR China; 3 Department of Psychiatry, Jining Medical College, Jining, Shandong Province, 272051, PR China; 4 Department of Psychiatry, Jining Mental Hospital, Jining, Shandong Province, 272051, PR China; 5 Department of Psychiatry, The Third Affiliated Hospital, Sun Yat-sen University, Guangzhou, Guangdong Province, 510630, PR China; 6 Institute of Mental Health, The Second Xiangya Hospital, Central South University, Changsha, Hunan Province, 410011, PR China; 7 Department of Neurology, First Affiliated Hospital, Sun Yat-sen University, Guangzhou, Guangdong Province, 510080, PR China; Huashan Hospital, Fudan University, CHINA

## Abstract

Parkinson’s disease (PD) and schizophrenia (SCZ) are frequent central nervous disorders that have unclear etiologies but that show similarities in their pathogenesis. Since elevated histamine levels in the brain have been associated with PD and SCZ, we wanted to explore whether the Thr105Ile substitution in the *histamine N-methyltransferase* gene (*HNMT-*Thr105Ile), which impairs histamine degradation, is associated with either disease. We used the ligase detection reaction to genotype a case-control cohort of Han Chinese patients with PD or SCZ and healthy controls at the *HNMT*-Thr105Ile locus. The Ile allele was associated with reduced risk of PD (OR 0.516, 95%CI 0.318 to 0.838, p = 0.007) and of SCZ (OR 0.499, 95%CI 0.288 to 0.865, p = 0.011). Genotype frequencies and minor allele frequencies were similar between patients and controls when we compared males with females or early-onset patients with late-onset ones. Genotype and allele frequencies were not significantly different between PD patients with dyskinesia and PD patients without dyskinesia. Our results suggest that the heterozygous Thr/Ile genotype at the *HNMT*-Thr105Ile locus and the minor Ile105 allele protect against PD and SCZ in Han Chinese.

## Introduction

Parkinson’s disease (PD) and schizophrenia (SCZ) are devastasting central nervous disorders and despite decades of research, their etiology remains unclear. However, numerous studies have documented similarities in the pathogenesis of both diseases. The risk of both PD and SCZ is higher in patients with the Val158Met polymorphism in the gene for catechol-O-methyltranferase (*COMT*)[1,2], the rs1799836 polymorphism in the gene for monoamine oxidase B (*MAOB*) [1,3], or the C(-1562)T polymorphism in the gene encoding matrix metalloprotease-9 (*MPP-9*) [4,5]. Dysregulation of several neurotransmitters, including dopamine and histamine, is associated with PD and SCZ[6,7]. In fact, substantial evidence indicates an association of the dopamine metabolism pathway with PD and SCZ, and the main treatments for PD and SCZ are based on the dopamine pathway [8,9].

Like the dysregulation of dopamine, dysregulation of histamine levels in the brain may be another unifying element in the pathogenesis of both PD and SCZ. Histamine can selectively damage dopaminergic neurons of the substantia nigra pars compacta (SNc), leading to the increased inflammation that is characteristic of PD pathology [10]. Patients with SCZ show lower density of histamine H1 receptors in the frontal, prefrontal, and cingulate cortex than do controls [11]. In addition, levels of the histamine metabolite tele-methylhistamine in the cerebrospinal fluid are 2.6-fold higher in SCZ patients than in healthy individuals, suggesting abnormally high histamine turnover [12]. Histamine H2 antagonist therapy has shown clinical benefits for patients with SCZ without significant adverse effects in a recent placebo-controlled randomized clinical trial[13]. The same type of therapy has proven effective at treating levodopa-induced dyskinesia in an animal model of PD and in patients with PD [6,14]. These findings suggest that histamine levels and histamine metabolism may influence onset and progression of PD and SCZ.

The enzymes histamine N-methyltransferase (HNMT) and diamine oxidase (DAO) degrade histamine, with HNMT playing the leading role in histamine metabolism in the central nervous system. The *HNMT* gene has even been proposed as a genetic biomarker for PD [15]. A sequence variant of the *HNMT* gene, exon 4(C314T), causes the amino acid substitution Thr105Ile in the enzyme, reducing its activity and increasing histamine levels in the brain [16]. This Thr105Ile mutation is the only functional mutation identified so far in the *HNMT* coding region in Chinese populations [17]. Several studies have examined a possible association between the *HNMT*-Thr105Ile polymorphism and PD, but the results have been inconsistent [18–20]. At the same time, we are aware of only one published study examining the association between *HNMT*-Thr105Ile and risk of SCZ, which found no association [21]. That study involved a relatively small sample of 185 patients with SCZ and 189 healthy controls, and the subjects came from several ethnicities; both factors may have significantly affected the outcome.

To help resolve the controversy over whether the *HNMT*-Thr105Ile variant is associated with risk of PD and SCZ, we performed a case-control study in a relatively large, single-ethnicity cohort of Han Chinese.

## Materials and Methods

### Materials

A total of 564 Han Chinese patients with sporadic PD (305 males, 259 females) were consecutively recruited from two movement disorder centers: West China Hospital of Sichuan University, located in southwest China; and First Affiliated Hospital of Sun Yat-sen University, located in southeast China. PD was diagnosed in all patients by two independent movement disorder specialists based on the UK Parkinson’s Disease Society Brain Bank criteria for idiopathic PD [22]. Patients with at least one relative with PD were excluded from the study. As controls, 496 healthy Han Chinese (294 males, 202 females) unrelated to the PD cohort were recruited. Average age was 62.75±12.84 yr for PD patients, and 61.91±11.51 yr for healthy controls (Table 1). Patients who were younger than 50 years at onset were classified as having early-onset PD (EOPD; n = 167; mean age at onset, 44.37±5.35 yr); others were defined as having late-onset PD (LOPD; n = 397; mean age at onset, 63.05±6.59 yr). Patients were further divided into those with dyskinesia (n = 124) and those without it (n = 440).

**Table 1:** Demographic data on Han Chinese with Parkinson’s disease (PD) or schizophrenia (SCZ) and healthy controls.

Factor	SCZ (n = 423)	Controls (n = 457)	Comparison*	PD (n = 564)	Controls (n = 496)	Comparison
Age, yr	36.11±13.61	36.69±10.86	t = 0.70; p = 0.48	62.7±12.84	61.9±11.51	t = 1.13; p = 0.26
Gender						
Male	180	193	p = 0.923	305	294	p = 0.089
Female	243	264		259	202	

Patients with SCZ were recruited from four centers: The Second Xiangya Hospital of Central South University, located in central China; West China Hospital of Sichuan University, located in southwest China; and the Third Affiliated Hospital of Sun Yat-sen University and Jining Mental Hospital, both located in east China. A total of 423 Han Chinese patients were recruited (180 males, 243 females; mean age, 36.11±13.61 yr). All patients were diagnosed by two psychiatrists based on the Structured Clinical Interview for DSM Disorders (SCID) and DSM-IV criteria [23]. Patients who were older than 18 at SCZ onset were classified as having late-onset SCZ (LOSCZ; n = 287; mean age at onset, 25.99±6.77 yr); others were classified as having early-onset SCZ (EOSCZ; n = 136; mean age at onset, 15.35±2.13 yr) [24]. The control group for SCZ patients comprised 457 Han Chinese (193 males, 264 females; mean age, 36.69±10.86 yr).

Control individuals were screened for mental disorders and for family history of mental disease. The corresponding controls were well-matched with SCZ patients in terms of age and gender. To take into account the different age distributions between our PD and SCZ groups, we recruited a healthy control population for the SCZ cohort that was entirely different from that of the PD cohort. Each control group was well-matched with PD or SCZ patients in terms of age and gender (Table 1).

The protocol of the study was approved by the ethics committees of West China Hospital of Sichuan University, the First Affiliated Hospital of Sun Yat-sen University, the Second Xiangya Hospital of Central South University, the Third Affiliated Hospital of Sun Yat-sen University, and Jining Mental Hospital. Written informed consent was obtained from all subjects.

### Genotyping

Genomic DNA was obtained from peripheral leukocytes by classical phenol- chloroform extraction. All genotyping was performed by the Shanghai BioWing Applied Biotechnology Company using the ligase detection reaction (LDR) [25]. Briefly, this method involved the following steps. Target DNA in the *HNMT* gene was amplified using a multiplex PCR method using the forward primer 5’-GCCAAGCA AACTTTACGTTC-3’ and the reverse primer 5’-TGATGGTGTGTCACCTCTTC-3’. Then amplifications were mixed with 1 μl of proteinase K (20 mg/ml), incubated at 70°C for 10 min and then at 94°C for 15 min. Ligation reactions (20 μl) were set up with 20 mM Tris-HCl (pH 7.6), 25 mM potassium acetate, 10 mM magnesium acetate, 10 mM DTT, 1 mM NAD, 0.1% Triton X-100, 10 μl of amplicon, 1 pmol of each discriminant primer, 1 pmol of each universal primer and 0.5 μl of 40 U/μl Taq DNA ligase (New England Biolabs, USA). Ligation was performed using 40 cycles at 94°C for 30 s and 63°C for 4 min. Fluorescent ligation products were analyzed on an ABI Sequencer 377.

Several measures were taken to ensure accurate genotyping. First, the technicians performing the genotyping were blinded to the case or control status of the samples. Second, a random selection of 20% of the samples was genotyped independently by other technicians; the results of this second round of testing were identical in all cases to the initial results. Third, we selected 10 samples for each variant genotype obtained in the ligase detection reaction and we sequenced them directly using an automated sequencer (ABI Prism 3730); in all cases, the expected sequences were obtained.

### Statistical analysis

All statistical analyses were performed using SPSS 17.0 (IBM, Chicago, USA). Age was reported as mean±SD, while gender, allele and genotype frequencies were reported as percentages. Allele and genotype frequencies were determined by direct counting of *HNMT* alleles. Concordance between genotype distributions was verified by comparison with the predictions of Hardy-Weinberg equilibrium (HWE); differences were assessed using the chi-squared test. Associations among gender, allele and genotype were assessed using the chi-squared test. Intergroup differences in age at the time of the study and in age at onset were assessed using the t test. A two-tailed P value < 0.05 was defined as the threshold of statistical significance.

### Result

The genotype distribution at the *HNMT-*Thr105Ile locus and frequencies of individual alleles in patients with PD and the corresponding control group are shown in Table 2. The genotype distribution was in accordance with HWE for patients (χ^2^ = 0.34, p = 0.56) and controls (χ^2^ = 0.0005, *p* = 0.98).The Thr/Ile+Ile/Ile genotype was significantly less frequent among patients than controls (OR 0.53, 95%CI 0.322 to 0.871, p = 0.013), as was the Ile105 allele (OR 0.516, 95%CI 0.318 to 0.838, p = 0.007). We also examined whether patients and controls differed significantly in genotype frequencies or minor allele frequency when we compared males with females, early-onset patients with late-onset ones or patients with dyskinesia and patients without dyskinesia. Frequencies were similar between patients and controls in all these subgroup analyses (Table 3).

**Table 2:** Polymorphism at the *HNMT*-Thr105Ile locus in Han Chinese patients with PD or SZ and in healthy controls.*

Variant	PD	Control	Comparison**	SCZ	Control	Comparison**
*Genotype*						
Thr/Thr	537 (94.2)	452 (91.03)	0.53;0.322–0.871; 0.013***	405 (95.7)	418 (91.47)	0.499;0.268–0.847; 0.010***
Thr/Ile	27 (4.8)	43 (8.76)		18 (4.3)	39 (8.53)	
Ile/Ile	0	1 (0.2)		0	0	
*Allele*						
Thr	1101	947	0.516;0.318–0.838;0.007	828 (97.75)	875 (95.73)	0.499;0.288–0.865; 0.011
Ile	27	45		18 (2.25)	39 (4.27)	

* Values for the PD, SCZ and control groups are reported as n (%).

**Unless otherwise indicated, the values refer to OR; 95%CI; P value.

***Ile/Ile+Thr/Ile vs Thr/Thr

**Table 3:** Distributions of minor allele frequency (MAF) in Han Chinese patients with SCZ or PD, stratified by gender or age of onset.

Subgroup	Genotype / MAF				Comparison*
	Total	Thr/Thr	Thr/Ile	Ile	
*Patients with SCZ*					
Males	180	170	10	10	0.571;0.221–1.478;0.243 a
Females	243	235	8	8	1.707;0.667–4.370;0.259 b
EOSCZ	136	128	8	8	1.731;0.668–4.490;;0.254 a
LOSCZ	287	277	10	10	1.709;0.667–4.380;;0.259; b
*Patients with PD*					
Male	305	293	12	12	1.501;0.690–3.267;0.303 a
Female	259	244	15	15	0.637;0.312–1.451;0.309 b
EOPD	167	161	6	6	0.637;0.312–1.451;0.309 b
LOPD	397	376	21	21	0.673;0.312–1.451;0.309 b
With dyskinesia	124	121	3	3	1.876;0.556–6.332;0.303 a
Without dyskinesia	540	516	24	24	1.856;0.554–6.213;0.308 b

*Values indicate: OR; 95%CI;P value. Results marked with “a” refer to the genotype distribution; results marked with “b” refer to MAF distribution.

The genotype distribution at the *HNMT-*Thr105Ile locus and frequencies of individual alleles in patients with SCZ and the corresponding control group are shown in Table 2. The genotype distribution was in accordance with HWE for patients (χ^2^ = 0.20, p = 0.65), and for controls (χ^2^ = 0.91, p = 0.34). The Thr/Ile genotype was significantly less frequent among patients than controls (OR 0.499, 95%CI 0.268 to 0.847, p = 0.010), as was the Ile105 allele (OR 0.499, 95%CI 0.288 to 0.865, p = 0.011). Genotype frequencies and MAF were similar between patients and controls in subgroup analyses based on gender or age at onset (Table 3).

### Discussion

Our results suggest that the *HNMT*-Thr105Ile locus is associated with risk of both PD and SCZ in Han Chinese, with the heterozygous genotype Thr/Ile and the minor Ile105 allele conferring a protective effect against both disorders. To the best of our knowledge, this is the first study to relate variations at the *HNMT*-Thr105Ile locus to PD and SCZ in an Asian population.

We detected the Ile/Ile allele in only one of 496 subjects in the control group matched to PD patients; a similarly low frequency was also reported in a previous study of *HNMT*-Thr105Ile polymorphism in Chinese [26]. The frequencies of the heterozygous genotype Thr/Ile and the Ile allele are significantly lower in our population than in European and North American populations [18]. However, PD and SCZ appear to be less prevalent among Asians than in these other populations [27,28], contrary to what one might predict if the Thr/Ile genotype and Ile allele protect against these disorders. This apparent paradox presumably reflects the strong influence of gene-gene and gene-environment interactions in determining the overall risk of disease.

Such influences may also help explain why we failed to detect significant differences in genotype distribution between PD patients with dyskinesia and PD patients without it, even though histamine H2 antagonist therapy has proven effective at treating levodopa-induced dyskinesia in an animal model of PD and in patients [6,14]. Since our findings are based on only 124 patients with dyskinesia, larger studies are needed to verify this result.

The *HNMT* gene, located at 2q22.1, encodes an enzyme that methylates histamine in the extracellular space of the central nervous system. Histamine is an important neurotransmitter in the brain, and HNMT-mediated methylation is the only way to deactivate it, since the mammalian brain lacks a histamine reuptake system [29]. Numerous lines of evidence suggest that histamine hypermetabolism is associated with the pathophysiology of PD. This hypermetabolism may involve increased synthesis to compensate for a relatively rapid deactivation [add here the Agundez et al. reference]. Elevated serum levels of histamine have been detected in patients with PD [30], and non-medicated patients with mild to moderate PD show elevated levels of the histamine metabolite pros-methylimidazoleatic acid in the cerebrospinal fluid[31]. Autopsy studies of patients with PD have revealed elevated levels of histamine in areas associated with motor behavior, including the caudate nucleus, putamen, internal and external globus pallidus and the SNc. Autopsy studies have also shown that histaminergic fibers, where the neurotransmitter is synthesized, are denser in patients with PD than in controls, and that a greater proportion of these fibers have enlarged varicosities, where histamine is stored [32]. Post-mortem studies have shown higher levels of *HNMT* mRNA in the SNc and putamen of patients than of healthy individuals, and the precise mRNA level may correlate with PD severity [33]. Administering an irreversible inhibitor of histamine synthesis to a rat model of PD produced significant protection against neuronal loss [34]. Histamine hypermetabolism may contribute to PD pathophysiology by inhibiting dopamine activity: in a rat model of PD, a selective H3 receptor agonist attenuated dopamine release in the striatum [35]. Together, the findings suggest that elevated histamine metabolism may promote PD onset and/or progression, perhaps by disturbing dopamine signaling, whereas reduced histamine metabolism may exert a protective effect. Consistent with this literature, we found in the present study that the minor allele Thr105Ile, which reduces HNMT-mediated histamine deactivation, helps reduce risk of PD. This may be because the reduction in deactivation causes a compensatory reduction in synthesis, which should be tested in future studies.

Whether SCZ involves a similar disruption of histamine homeostasis is unclear, but several pieces of evidence point in that direction. Autopsy studies of patients with SCZ show that the density of histamine H1 receptors in the frontal cortex is lower in patients with SCZ than in controls [11]. In addition, levels of histamine metabolites in the cerebrospinal fluid are higher in patients with SCZ, suggesting histamine hypermetabolism similar to that in PD[12]. It may be no coincidence that antipsychotic drugs such as clozapine and olanzapin act, in least in part, through histamine receptors [36]. Indeed, a new generation of non-dopaminergic drugs to treat both PD and SCZ bind to histamine H2 receptors[6,13].

Our results provide strong evidence that the *HNMT* gene is associated with PD and SCZ, with a power of 0.955 for the PD association and 0.912 for the SCZ association. These findings extend the list of diseases already associated with the gene, including alcoholism [37], essential tremor [38], allergic rhinitis [39], asthma [40], and myasthenia gravis [41]. Future studies should explore to what extent histamine dysregulation is important in the onset or progression of these disorders.

Our observation of an association between the *HNMT*-Thr105Ile polymorphism and PD risk is consistent with previous studies in Caucasians from Spain [16] and from Europe and the US [20], but at least one study, on Caucasians from North America, concluded that there was no association [19]. These discrepancies may reflect strong influence of different genetic backgrounds in different ethnicities. It may also reflect differences in gene-gene or gene-environmental interactions. The *HNMT*-Thr105Ile polymorphism would not be the first to show different effects on PD risk as a function of ethnicity or environment. The rs11724635 polymorphism in the *BST1* gene increases the risk of PD to a significantly greater extent in Asians than in Caucasians. Another *BST1* polymorphism, rs11724635, is not by itself associated with PD in ethnic Taiwanese, but when carriers drink well water, it does increase the risk of the disease[42]. Future studies should examine the extent to which other genes and environment may affect the influence of the *HNMT*-Thr105Ile polymorphism on PD and SCZ.

The results of our study should be interpreted with caution given its limitations. Although we examined a relatively large study population, the low minor allele frequency reduced the overall statistical power. In addition, we focused on the one functional SNP in the *HNMT* gene, leading us to neglect SNPs in other regions of the gene that may be playing a disease role. Thus, future studies should examine more *HNMT* variants in a larger study population in order to gain a more complete picture of the potential association of this gene with PD and SCZ.

In conclusion, our multi-center study shows that the *HNMT*-Thr105Ile polymorphism is associated with PD and SCZ in Han Chinese. Nevertheless, these associations should be verified in larger studies of other ethnicities. Future studies are also needed to understand how *HNMT* polymorphism affects histamine homeostasis, and how histamine dysregulation contributes to PD, SCZ and potentially other motor disorders.
